# Phase separation in lead-saponified drying oils: Implications for historical painting techniques and paint stability

**DOI:** 10.1126/sciadv.adt0897

**Published:** 2025-08-27

**Authors:** Lucie Laporte, David Touboul, Thierry Pouget, Nicolas Benoot, Guylaine Ducouret, Sophie Rochut, Maguy Jaber, Frédéric Gobeaux, Laurence de Viguerie

**Affiliations:** ^1^ LAMS (Laboratoire d’Archéologie Moléculaire et Structurale), CNRS UMR 8220, Sorbonne Université, 4 Place Jussieu, 75005 Paris, France.; ^2^ CY Cergy Paris Université, LPPI (Laboratoire de Physicochimie des Polymères et des Interfaces), 5 mail Gay Lussac, 95000 Neuville sur Oise, France.; ^3^ LCM (Laboratoire de Chimie Moléculaire), CNRS, Ecole Polytechnique, Institut Polytechnique de Paris, 1120 Palaiseau, France.; ^4^ Materials Innovation Department, LVMH Research, Helios Research Center, 45800 Saint-Jean-de-Braye, France.; ^5^ Soft Matter Science and Engineering (SIMM), ESPCI Paris, PSL University, Sorbonne Université, CNRS, F-75005 Paris, France.; ^6^ LIONS – NIMBE (Interdisciplinary Laboratory on Nanoscale and Supramolecular Organization - Nanosciences and Innovation for Materials, Biomedicine and Energy), CEA/CNRS UMR 3685, Université Paris-Saclay, CEA Saclay, 91191 Gif sur Yvette, France.

## Abstract

Renaissance Masters often prepared siccative oils by heating linseed oil with siccatives, particularly lead oxide, inducing partial saponification and altering its properties. Our reconstructions show that lead-saponified oils naturally separate into two phases. In this study, we investigate the differences between these two phases through a comprehensive set of analytical methods, from macrolevel assessments (rheology) to microlevel characterizations (small and wide-angle x-ray scattering, optical microscopy, and scanning electron microscopy) and chemical analyses. The lower phase is enriched in free fatty acids and lead carboxylates, especially saturated species—both as free acids and metal soaps—prone to self-organization. As a result, the lower phase displays a pronounced lamellar organization with partial crystallization and exhibits viscoelastic and shear-thinning properties. In contrast, the unstructured upper phase behaves as a Newtonian fluid. The observed phenomena share similarities with the formation of soap-related defects such as protrusions, linked to saturated lead soaps.

## Teaser

Spontaneous phenomenon of phase separation in saponified oils influences the structure and behavior of historical paint systems.

## INTRODUCTION

In the 15th century, oil painting techniques saw unprecedented advancements in Flanders and Italy, driven by innovations in the drying properties of oil. Pioneering artists, notably the Flemish painter J. Van Eyck, revolutionized oil binders by heating them with siccative compounds, also known as driers (*1*). Historical painting treatises provide extensive documentation of this practice, detailing the heating of oils with metallic driers that accelerate oil curing and the formation of a stable film (*1*, *2*). Among the most frequently mentioned driers was litharge, or lead oxide (PbO) (*3*). This technique persisted through centuries and was later adapted into industrial processes in the 20th century. By then, the standard practice involved allowing the oil to settle, using only the “clear and transparent oil” (*4*). While Renaissance recipes do not mention it, these treated oils exhibit long-term instability, resulting in a phase separation or “demixing” into two distinct phases. This separation occurs not immediately after heating but in the following days or even within hours if water is added during the heating process.

To better understand how this phase separation may have influenced the painting techniques of the Old Masters and the conservation of their artworks, it is crucial to investigate the nature and chemical behavior of these two phases. This requires linking their chemical composition to their supramolecular organization and macroscopic properties.

From a chemical perspective, siccative oils used in paints (such as linseed, poppy, or walnut oils) are primarily composed of triglycerides, with aliphatic chains that are predominantly polyunsaturated, specifically linoleic (C18:2) and linolenic acids (C18:3). In addition, about 15% of the fatty acids are either saturated (palmitic acid C16:0 and stearic acid C18:0) or monounsaturated (oleic acid C18:1) (*5*). When PbO is introduced to the oil, partial saponification of triglycerides occurs, forming long-chain carboxylates (soaps) from the aforementioned aliphatic chains, along with other minor reactions such as oxidation, isomerization, and cleavage (*6*, *7*). As a result, the binder becomes a complex mixture of lead carboxylates, glycerol, mono-, di-, and triglycerides, as well as oxidized and isomerized products. Several studies have explored the chemical composition of saponified oils, prepared with or without water (often included in recipes to prevent oil darkening) (*2*), investigating their chemical composition (*8*), their saponification kinetics (*9*), their flow properties (*10*), and, more recently, the architecture of lead soaps at the micro- and mesoscale (*11*, *12*). Lead soaps have also been identified in degraded areas of aged oil paintings, such as protrusions of saturated soaps (*13*, *14*), and the use of PbO-based saponified oils has been shown to promote the formation of degradation compounds, such as plumbonacrite (a lead hydroxycarbonate) during paint aging (*15*, *16*).

In this study, we use a suite of complementary techniques to refine our understanding of the phase separation of the lead-treated oils. First, we correlate the rheological properties of the two distinct phases with their structural organization at the nanometric [small-angle x-ray scattering (SAXS) and wide-angle x-ray scattering (WAXS)] and micrometric (optical and electron microscopy) scales. These methods complement each other: X-ray scattering reveals the specific organization of lead soaps, with a signal dominated by the elastic scattering of lead atoms, while microscopy provides a broader perspective on the overall structuring of the model systems (soaps and oil matrix). We then establish links between the structure and chemical composition of the saponified oils. Given the complexity of partially saponified oils, we enhance nonselective characterization techniques [attenuated total reflection–Fourier transform infrared (ATR-FTIR) and thermogravimetric analysis (TGA)] with chromatographic methods tailored to specific oil components, such as triglycerides [supercritical fluid chromatography coupled to high-resolution mass spectrometry (SFC-HRMS)] and fatty acids or soaps [gas chromatography–mass spectrometry (GC-MS)]. This innovative combination of analytical approaches yields a detailed fingerprint of the two phases, offering critical insights into the mechanisms underlying phase separation. By describing the two phases at multiple scales, we reveal how the structuration of saturated lead soaps affects their supramolecular organization and rheological properties.

## RESULTS

### Macroscopic properties of saponified oil after phase separation

We focus on the saponified oils prepared with water, for which we observed a pronounced demixing after only a few hours. Systems were prepared according to written sources of the 16th to 17th century (*2*) translated into a reproducible chemical protocol (see experimental details in Materials and Methods). In particular, we followed the recommendation of the manuscript by de Mayerne *et al.* (*2*), indicating that some unevaporated water should be left at the end of the heating process and that this supernatant water should be removed: [...] “remove from the fire, then separate the oil from the water, and keep it for your own use.” To prevent from the complete evaporation of water during the heating process (and subsequent darkening), we introduced the same weight of water as oil. Ten hours after the end of heating, two phases are visible; centrifugation is carried out after 24 hours to separate them efficiently (fig. S1) and eventually remove the remaining supernatant water.

**Fig. 1.**
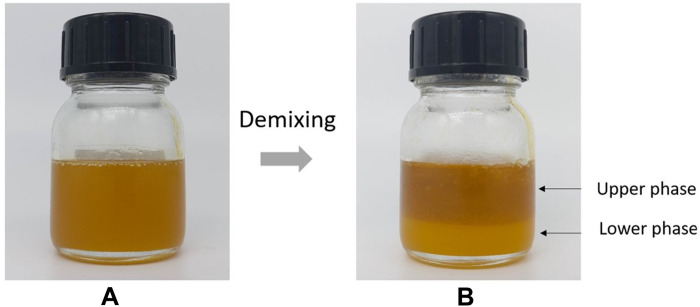
Demixing of saponified oils. Aspect of the oil prepared by heating linseed oil and PbO (50 mol %, i.e., 20 wt %) with water (**A**) just after preparation and (**B**) after 2 days.

**Fig. 2.**
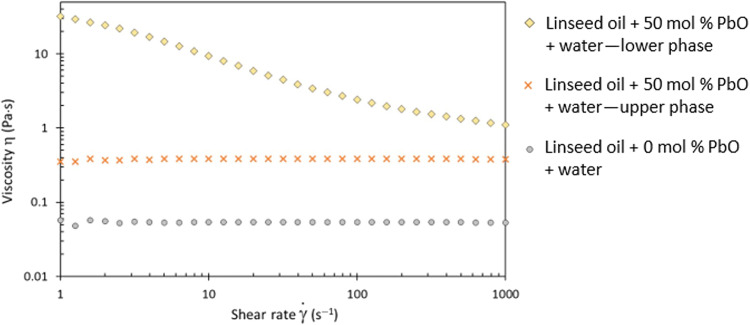
Comparison of rheological properties of the two phases. Flow tests from 1 to 1000 s^−1^ on linseed oil heated with water and linseed oil + 50 mol % PbO heated with water after centrifugation. The same flow test of linseed oil heated with water, without PbO, is plotted for comparison. Data obtained for oils containing 17 and 31 mol % PbO are shown in fig. S2.

Visually, the lower phase is pasty, opaque, and pale yellow in color, while the upper phase is fluid, transparent, and orange-yellow (Fig. 1). Their rheological properties were characterized: The lower phase displays shear-thinning behavior, whereas the upper phase is completely viscous and behaves as a Newtonian fluid (Fig. 2). This difference in behavior was observed for all investigated formulations, i.e., at 17, 31, and 50 mol %, corresponding to 5, 10, and 20 wt % (fig. S2). The lower phases also display strong viscoelastic properties, with an elastic modulus *G*′ greater than the loss modulus *G*″ at low strain amplitude (fig. S3) and over the entire frequency range tested (fig. S4). These elastic properties and shear-thinning behavior point toward a structuration of the lower phase, destroyed during shearing.

### Comparison of supramolecular organizations

**Fig. 3.**
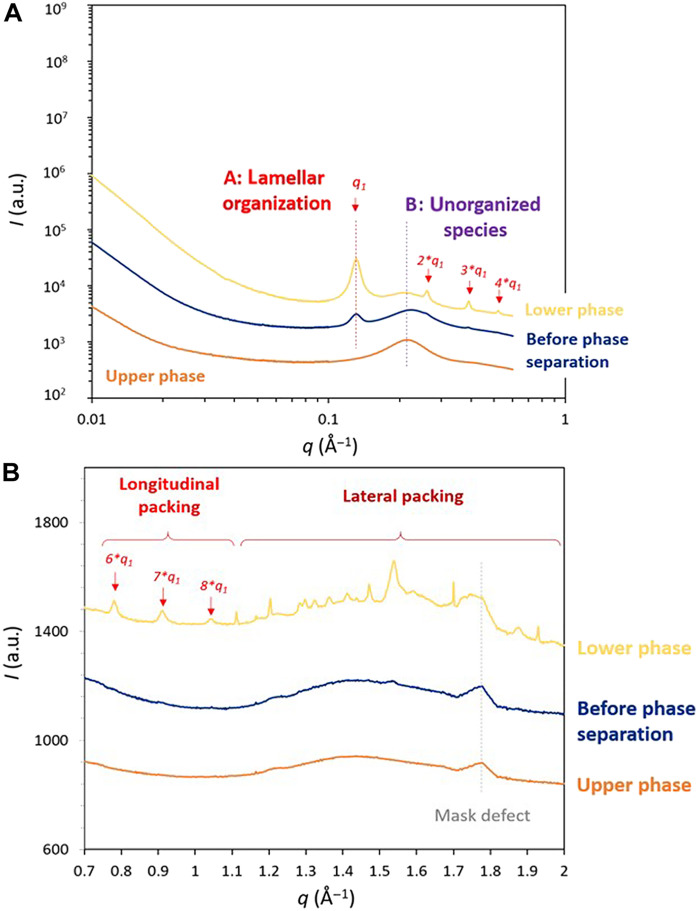
Comparison of the SAXS and WAXS signals of the two phases. Scattered intensity profiles as a function of the scattering vector *q*, from (**A**) 0.002 to 0.6 Å^−1^ (SAXS) and (**B**) 0.7 to 2 Å^−1^ (WAXS) for linseed oil + 50 mol % PbO heated with water, before and after phase separation. The two contributions observed on the SAXS profiles of saponified phases are noted A (lamellar organization, in red) and B (liquid order, in purple). Red arrows indicate periodic peaks characteristic of a local lamellar organization, with *q*_1_ as the position of the first peak. Data were obtained under synchrotron beam (SWING, Synchrotron SOLEIL, Gif-sur-Yvette). The signal at 1.76 Å corresponds to a mask defect.

To probe the structural properties of the two phases, SAXS and WAXS were then carried out before and after phase separation. The SAXS profile of saponified linseed oil is dominated by the elastic scattering of lead atoms, which have a longer elastic scattering length than the other atoms in the system (mainly C, H, and O). SAXS analyses revealed that the lower phase exhibits a highly ordered structure (Fig. 3A for 50 mol % PbO and fig. S5, A and B, for 17 and 31 mol % PbO): The corresponding scattered intensity profile shows a series of periodic peaks indicating a pronounced organization of lead soaps into lamellar structures. A similar lamellar organization was previously observed in saponified oil before demixing (*12*), where some lead soaps form microscopic lamellar domains dispersed within a continuous matrix of unorganized species. In the lower phase, the lamellar period (*d* = 48 Å), defined as

d=2πq1 (1)

(*q*_1_ being the position of the first-order peak), is close to the long spacing of lead stearate (50 Å). Although this value remains consistent with the system before separation, higher harmonics are now visible (up to eight, according to WAXS), and the full width at half maximum (FWHM) of the periodic oscillations is narrower. According to the Scherrer Eq. 2, the FWHM can be used to estimate the correlation length of the lamellar domains ξ (in angstrom) (*17*)

ξ=2×KFWHM (2)

where *K* is a constant related to the shape of the lamellar domains, fixed at 0.9 for undefined shape (*18*). Using this, the estimated size of the lamellar domains in the lower phase is about 750 Å, compared to 550 Å before phase separation (fig. S5C). In addition, the presence of numerous harmonics in the lower phase suggests reduced flexibility of the lamellae (*12*, *19*–*21*). Overall, the lower phase displays a more extensive lamellar organization and greater rigidity than the system before separation. The scattering profiles also show a broad liquid-order signal centered at 0.23 Å^−1^ (noted as B, in purple), indicating that both phases, including the structured lower phase, contain unorganized lead soaps, with a characteristic distance between lead centers of *d*_B_ = 28 Å.

WAXS data (Fig. 3B) offer further insights into the lateral packing of the organized lead soaps (short-spacing distances) within the lower phase. A series of sharp peaks indicates a high proportion of soaps with an ordered lateral arrangement, whereas only a broad peak is visible between 1.1 and 1.9 Å^−1^ for both the noncentrifuged sample and the upper phase. Although the lamellar period measured by SAXS aligns with the long spacing of lead stearate (50 Å), no perfect correspondence is found in the WAXS signal for the characteristic lateral packing of soaps (whether lead stearate, lead palmitate, or lead oleate) (*22*). This lamellar organization likely arises from a mixture of saturated and unsaturated soaps and/or from soaps with a lower degree of order than that of a fully crystallized phase, as described in the mesophases by Martínez-Casado *et al.* (*23*).

**Fig. 4.**
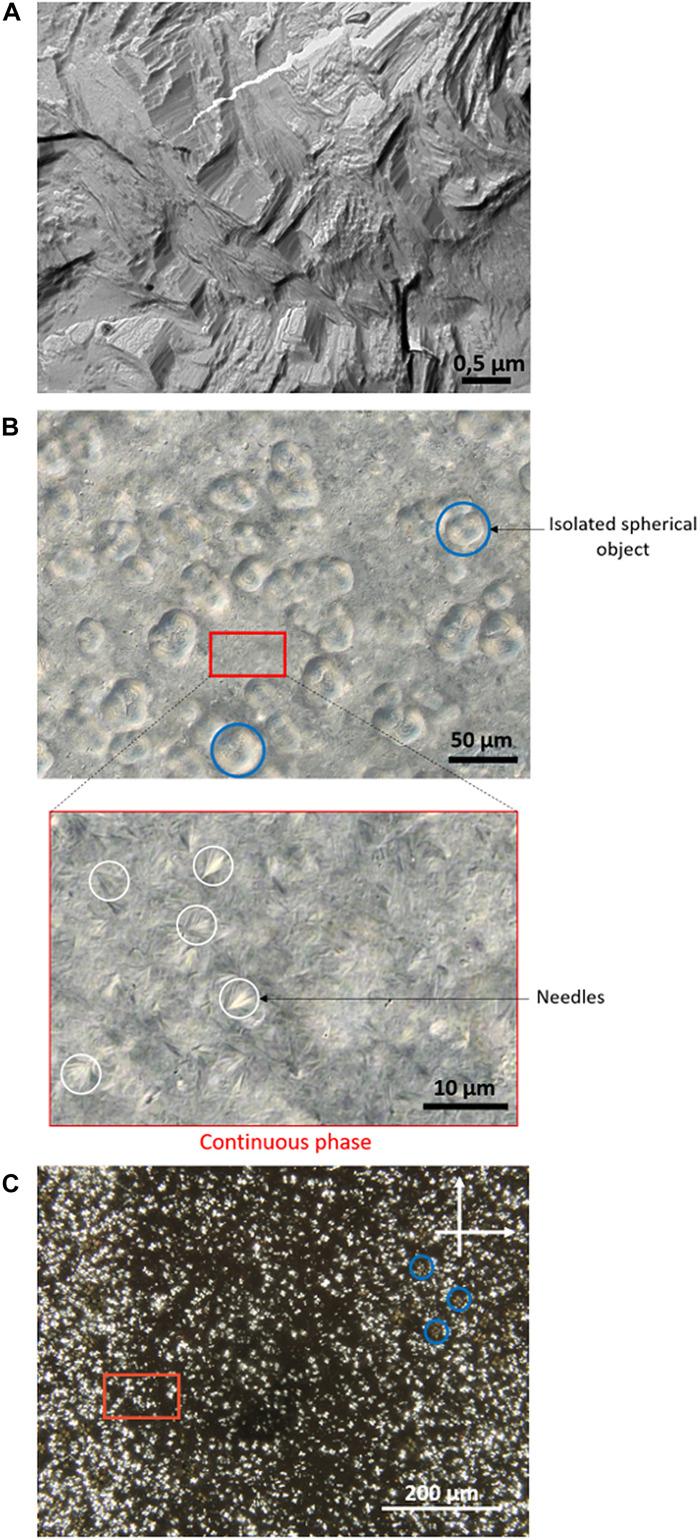
Microscopy observations of the lower phase. (**A**) FF-TEM, (**B**) DIC microscopy, and (**C**) polarized light optical microscopy (PLOM) images of the lower phase of linseed oil + 50 mol % PbO heated with water. DIC images reveal spherical objects (circled in blue) dispersed in a continuous phase (boxed in red) composed of needle-like spherulites (circled in white). Both types of objects are also visible in PLOM.

Complementary microscopy observations were carried out on the lower phase. Freeze fracture transmission electron microscopy (FF-TEM) revealed that the lamellar domains are aggregated and fractured in multiple directions (Fig. 4A). No continuous phase is visible, and the lamellar domains consist of numerous stacked lamellae. Differential interference contrast (DIC) microscopy, applied to the same sample, provided a micrometer-scale view of the lamellar domain organization, complementing the SAXS data. The lower phase appears fully structured, showing spherical objects with diameters ranging from 15 to 40 μm, alongside a juxtaposition of fine, medium-sized needle-like structures measuring 6 to 10 μm, embedded within a continuous phase (Fig. 4B). The objects observed within this continuous phase resemble spherulites, composed of highly ordered lamellae, growing radially from a central core (*24*). These structures have been previously identified in triglyceride systems (*25*, *26*), supporting the conclusion that most compounds in the lower phase exist in an ordered state. Parallels can also be drawn with the spherical structures described by MacDonald *et al.* (*27*), in complexes formed by reaction between ethyl linoleate and M^*n*+^(CH_3_COO)*n* (M = Pb^2+^, Zn^2+^) as a model for linseed oil paint, although the observed size is not the same (5 μm versus 15 to 40 μm in our case), supporting the hypothesis that the observed structures incorporate both saturated and unsaturated chains.

Polarized light optical microscopy (PLOM) further revealed that the lower phase is uniformly birefringent (Fig. 4C): At higher magnification, under polarized light, both types of objects described with DIC (isolated spherical particles and spherulites consisting of needles) exhibit birefringence. In contrast, the upper phase of the same sample appeared completely isotropic.

### Investigation of the chemical composition of the two phases

**Fig. 5.**
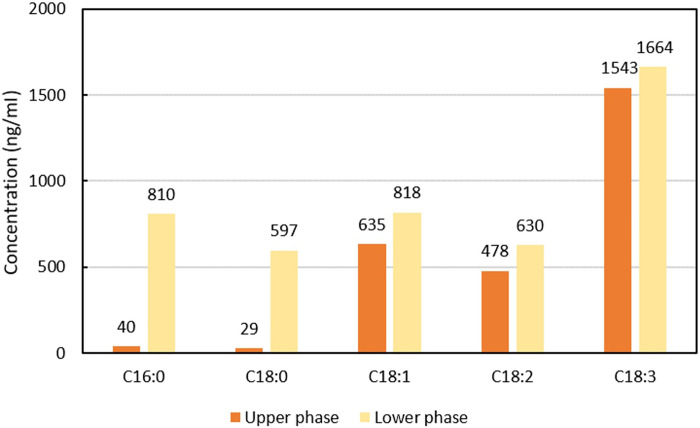
Comparison of the fatty acids composition (free and metal bound) of the two phases. GC-MS results for the absolute quantification of palmitic (C16:0), stearic (C18:0), oleic (C18:1), linoleic (C18:2), and linolenic (C18:3) acids in both phases of linseed oil + 50 mol % PbO heated with water. Quantifications have been performed using calibration curves from 2 to 1000 ng/ml after sample derivatization (fig. S8).

To better understand the differences between the two phases, their chemical composition was then investigated combining analytical techniques. Since water was used in the preparation of the systems, Karl-Fischer experiments were first conducted to measure the residual water content, which was found to be around 0.2 mol % in both phases. ATR-FTIR spectra (fig. S6) confirmed the presence of lead soaps [indicated by the asymmetric stretching band of lead carboxylates (ν_AS_ (C═O)] and suggested that their proportion is higher in the lower phase, at least for the lowest PbO initial content (17 mol % PbO). SFC-HRMS was used to provide an overview of the sample composition. This analytical method proved to be efficient for lipidomics studies in the biological (*28*, *29*) or food (*30*) fields, leading to the separation of lipids by classes for fast and efficient fingerprints when coupled to MS. Despite its potential, it remains underused in cultural heritage science. The acquired profile indicated that the distributions of tri- and diglycerides in the lower and upper phases are similar to the linseed oil reference, although the overall signal of triglycerides is lower in the lower phase (fig. S7 and table S1). During this analysis, lead soaps are not detected: Interactions between fatty acids and lead are probably lost during the sample preparation (dissolution in organic solvents) and surely lost during the chromatographic step where the salts are dissociated. To address this limitation, we thus carried out a complementary analysis using GC-MS, after derivatization with BSTFA [*N*,*O*-bis(trimethylsilyl)trifluoroacetamide]. It is known that BSTFA does not derivatize ester-bound lipids such as the one present in tri-, di-, and monoacylglycerols. In our case, the derivatization reaction will target both lead soaps and free fatty acids (and not tri- and diglycerides) (*31*). Absolute quantification (due to calibration curves; fig. S8) indicates that the total concentration of lead soaps and free fatty acids is higher in the lower phase. Saturated free and metal-bound saturated C16:0 and C18:0 fatty acids are mainly concentrated in the lower phase (Fig. 5).

TGAs were also performed to identify additional differences that could explain the observed structural variations. However, rather similar profiles were obtained (fig. S9), and the probable presence of higher molecular species in the lower phase could not be clearly assessed. Complementary differential scanning calorimetry (DSC) measurements indicated an endothermic phase transition at 70°C in the lower phase, whereas no such transition was detected in the upper phase, where all molecules remained in the liquid state (fig. S10). Although it was not possible to attribute this transition to any specific pure compound, the result aligns with the predominance of saturated chains, as the melting point of a fatty acid chain increases with chain length and decreases with unsaturation. It is also worth noting that unsaturated compounds in the trans conformation have higher melting points than their cis counterparts (*32*). A higher prevalence of trans isomers in the lower phase could potentially explain both the observed transition temperature and the greater structural organization of this phase, although this hypothesis could not be verified by other experimental characterizations.

## DISCUSSION

Saponified oils evolve into heterogeneous systems after formulation, with their components unevenly distributed. GC-MS, along with supporting data from SFC-HRMS, ATR-FTIR, and DSC, indicated that the lower phase is more concentrated in free fatty acids and lead carboxylates in particular. Notably, this phase also shows a substantial enrichment in saturated species. This variation in chemical composition results in a pronounced structural difference between the two phases, saturated lead soaps being more prone to self-organization: A well-defined lamellar structure is observed in the lower phase (as observed via SAXS and WAXS), with partial crystallization (evidenced by microscopy), while the upper phase remains unstructured. This structural disparity between both phases leads to distinct rheological behaviors: The lower phase exhibits viscoelastic, shear-thinning properties, whereas the upper phase behaves as a viscous, Newtonian fluid.

The phase separation is probably initiated by the progressive structuration of lead carboxylates, which gradually form organized micrometer-scale domains resembling spherulites [as observed in McDonald *et al.* (*27*)], probably corresponding to mixtures of saturated and unsaturated species, as evidenced by WAXS data, which reveal lateral packing characteristics similar to but distinct from those of saturated lead carboxylates. Factors such as a higher proportion of lead soaps and a greater fraction of high–molecular weight compounds could both contribute to sedimentation. However, while dimer or oligomer formation has been demonstrated in the literature under similar heating processes (*8*), the analytical techniques used in this study were not able to confirm their presence, leaving open the possibility that other methods might reveal them.

The described phase separation is a systematic phenomenon within the processes outlined in Materials and Methods. It occurs both with and without water addition during the heating process but proceeds faster when water is added. As water is completely removed by the end of the process (as confirmed by Karl-Fischer measurements), its role in the kinetics of phase separation may relate to the overall viscosity of the system: The addition of water during heating promotes agitation of the mixture and reduces the viscosity of the reaction medium. This reduction enhances species mobility, facilitating the segregation and nucleation of lead soaps (saturated, monounsaturated, and mixed) into well-ordered lamellar phases. This hypothesis aligns with Mills’ observation that the addition of a solvent accelerates phase transitions (*4*).

Considering artistic practices, it seems that the removal of the lower phase has only minimal impact on paint consistency and workability (on the basis of preliminary experiments that should be further developed). It is therefore plausible that painters were unaware of the significance of this phase separation, which might also explain why it is not explicitly described in historical treatises referencing these recipes. However, whether the binder was rehomogenized before adding pigments, or whether the lower phase was intentionally discarded remains closely tied to historical artistic practices and could have important implications for the evolution and conservation of artworks. If only the clear and transparent oil was used after the oil was allowed to settle, as Mills recommends for industrial saponified oil production (*4*), then the resulting mixture would be less rich in saturated lead soaps. This would directly affect the paint’s behavior during drying: Using only the upper phase would help painters avoid saturated lead soaps, which are prone to self-organization and have been linked to paint defects, such as the formation of protrusions. Those crystalline aggregates of saturated lead soaps (stearate, palmitate, and azelate) can appear years or centuries after completion of the painting (*13*, *14*, *33*). Preparing a paint with oil containing a lower amount of saturated lead soaps and no structuration in bulk would help in preventing from the formation of these drying defects. To validate this hypothesis, future research should monitor these formulations as paint layers to assess their propensity for forming protrusions. The removal of the lower phase, richer in saturated soaps, could substantially mitigate this risk.

The addition of water during the heating process, already recognized for its role in preventing darkening and ensuring homogeneous, stable heating, also accelerates phase separation. This practice (*34*) could further help painters avoid the complications linked to saturated lead soaps, thereby contributing to the long-term preservation of their works.

## MATERIALS AND METHODS

### Preparation of the lead-saponified oils

The used linseed oil was untreated cold pressed oil from Kremer (reference 73020). The composition of fatty acids is given by the provider as 61.3% linolenic acid, 14.6% linoleic acid, 15.3% oleic acid, 2.9% stearic acid, 4.4% palmitic acid, and other minor compounds (0.1% arachidic acid, 0.1% gadoleic acid, 0.2% lignoceric acid, and 0.3% nervonic acid).

Formulations containing 0, 5, 10, and 20 wt % PbO (corresponding respectively to 17, 31, and 50 mol % PbO) were prepared. The total weight of linseed oil and PbO was set at 20 g. The weight of ultrapure water added was the same as that of the oil. This ensures that nonevaporated water remains at the end of the heating process, as specified in the manuscript by de Mayerne *et al.* (*2*). Lead(II) oxide PbO (EMSURE) was first preground with 2 ml of linseed oil in a porcelain mortar. The remaining oil and the water were then added, and the mixture was transferred to a 100-ml round-bottom flask and heated in a silicone oil bath at a control temperature of 100°C for 180 hours, with magnetic stirring. The treated oil was then poured into a glass pillbox and left to cool at ambient temperature, and the supernatant water was removed with a glass pipette. Twenty-four hours after heating, the sample was centrifuged for 10 min at 10,000 rpm using a Sigma 2-16P centrifuge, to ensure reproducible separation of the two phases and thus facilitate their characterization. Two phases were obtained for all formulations, with the exception of heated oil without PbO, which remained homogeneous after centrifugation.

### Rheology

Rheology measurements were carried out on a HAAKE MARS 40 controlled stress rheometer (Thermo Fisher Scientific), equipped with sand-blasted stainless-steel cone-plate geometries (diameter, 20 and 35 mm; angle, 2°; gap, 0.104 mm). Flow tests were performed at 25°C, the temperature being maintained by an air Peltier module. Each measurement was repeated at least twice to check reproducibility. Shear rate sweep tests were then performed from 0.01 to 1000 s^−1^. Strain sweep tests from 0.05 to 1000% strain were then carried out on fresh samples at *f* = 1 Hz to evaluate the extent of the linear regime. When samples were viscoelastic, a frequency sweep test from 0.1 to 20 rad/s was performed in the linear viscoelastic plateau at constant strain of 0.1% on a third sample.

### SAXS and WAXS

Two different devices have been used: The SAXS and WAXS data for linseed oil + 50 mol % PbO before and after phase separation (Fig. 3) were acquired at the Synchrotron SOLEIL on the SWING beamline (BAG no. 20201118); the samples were irradiated by a 12-keV beam. The scattered intensity was collected by an EigerX4M detector (Dectris). Two sample-detector distances of 0.5 and 6 m were used, leading to an accessible *q* range of 0.002 to 0.6 Å^−1^ (SAXS) and 0.02 to 2 Å^−1^ (WAXS) after mask application. The obtained images were treated with Foxtrot 3.5 using an angular integration from 0 to 360°. Further details on sample preparation and data treatment can be found elsewhere (*12*). Scattered intensity profiles of phases with different PbO concentrations (fig. S5) were obtained with a Xeuss 2.0 (Xenocs) laboratory instrument from LIONS-NIMBE (CEA, Saclay, France) at a wavelength of 1.54 Å (Cu Kα source). The displayed *q* range (0.02 to 0.16 Å^−1^ after mask application) was achieved with a single sample-to-detector distance of 54 cm.

### FF-TEM, DIC microscopy, and PLOM

Following procedures previously described in (*12*), FF-TEM samples were frozen directly in liquid propane cooled to liquid nitrogen temperature and knife fractured at −125°C, under vacuum (1.6 × 10^–6^ mbar) using a BAF060 device from Leica. The cut surface was then coated with a layer of 4-nm platinum and 40-nm carbon. The resulting replicas were washed with a 50:50 mixture of tetrahydrofuran and chloroform to remove the sample underneath and then transferred on copper TEM grids (AGS160-4 carbon-coated grids Cu 400, from Oxford Instruments). Observations were performed on a CM 120 FEI TEM (Thermo Fisher Scientific) equipped with a LaB_6_ (lanthanum hexaboride) source, at an acceleration voltage of 120 kV. The images were collected using a charge-coupled device camera (resolution, 1300 by 1030), from Gatan, with an exposure time of between 0.3 2 s. The images were acquired and processed using digital micrograph software suite from Gatan.

DIC microscopy observations were performed in transmission between slide and coverslip on an Axio Imager 2 microscope (Zeiss) with a white light source. PLOM observations were carried out using a BX41 microscope (Olympus) equipped with a 12.5-million-pixel DP70 digital camera, polarizer, and analyzer. Liquid and pasty samples were observed between slide and coverslip.

### Karl-Fischer

Measurements were performed with a Karl-Fischer C20 coulometer (METTLER TOLEDO) in a glove box under inert gas (N_2_). The electrolyte was a methanolic solution of sulfur dioxide and imidazole. The end point of the titration was detected with a double platinum electrode. Each sample was titrated three times, and 0.1 g of oil was collected and injected for one measurement. A blank measurement was done between each measurement. To avoid any effect related to the aging of samples, treated oils were stored at 4°C in sealed pillboxes under inert gas (N_2_).

### Attenuated total reflection–Fourier transform infrared

ATR-FTIR measurements were carried out at room temperature using a Cary 630 spectrometer (Agilent Technologies), equipped with a single-reflection diamond ATR sensor and MicroLab acquisition software. Each sample was analyzed three times, with an accumulation of 256 scans and a resolution of 4 cm^−1^. Spectra were treated with Omnic 9.2.86 software (Thermo Fisher Scientific).

### Supercritical fluid chromatography coupled to high-resolution mass spectrometry

Samples were prepared at 1 mg/ml in isopropanol and diluted 1:100 before injection, so their final concentration was 10 μg/ml. The method was adapted from that developed by Chollet *et al.* (*28*). Analyses were performed on an SFC 1260 Infinity system (Agilent Technologies) equipped with a Torus DEA column (150 mm by 2.1 mm by 1.7 μm; Waters). The SFC system was coupled to an Agilent 6540 (Agilent Technologies) high-resolution tandem quadrupole-time-of-flight mass spectrometer equipped with an electrospray ionization system. Column temperature was set at 60°C. A methanol/ethanol mixture (50/50) with 20 mM ammonium acetate was used as cosolvent at a flow rate of 0.9 ml/min with the following gradient: 0.0 to 1.5 min (99 to 96%), 1.5 to 2.5 min (96 to 85%), 2.5 to 5.5 min (85%), 5.5 to 7.5 min (85 to 70%), 7.5 to 8.5 min (70 to 58%), 8.5 to 15 min (58%), and 15 to 16 min (58–99%). To improve analyte solubility at the column outlet and thus facilitate ionization, a mixture of methanol, ethanol, and ammonium acetate was added to the mobile phase [methanol/ethanol, 1:1 (v/v) + 20 mM CH_3_COONH_4_]. Last, to prevent CO_2_ condensation, a Caloratherm heater (Sandra Selerity Technologies) was placed in front of the ionizing source (*T* = 60°C). MS analyses were performed in positive-ion mode over the 50 to 1700 mass/charge ratio (*m/z*) range, with a scan rate of 2 spectra/s. The use of a calibration solution, containing two internal reference masses {purine (C_5_H_4_N_4_, *m/z* 121.0509) and HP-921 [hexakis-(1*H*,1*H*,3*H*-tetrafluoropentoxy)phosphazene] (C_18_H_18_O_6_N_3_P_3_F_24_, *m/z* 922.0098)}, routinely led to mass accuracy below 3 parts per million.

### Gas chromatography–mass spectrometry

Samples of saponified oils were weighed into a flask, and successive dilutions in heptane were performed to obtain a concentrated solution (40 μg/liter). Twenty microliters of a BSTFA solution (Supelco) were added to 150 μl of the diluted solution, and the mixture was heated at 90°C (±5°C) for 80 min. The use of BSTFA as a derivatizing agent allows probing specifically the distribution of the fatty acid chains from soaps and free fatty acids. The resulting solution was delivered to the injector of an Agilent 7890B-GC system (Agilent Technologies) coupled to an Agilent MSD 5977B single quadrupole mass spectrometer (Agilent Technologies). Volatile compounds were separated with a HP-5MS column (30 m by 0.25 mm by 0.25 μm; Agilent Technologies) subjected to the following temperature gradient: 80°C for 2 min and then 15°C/min to 280°C, followed by an isotherm at 280°C for 10 min. For all samples, the injection was performed in splitless mode. The injection volume was 1 μl, the injector temperature was set at 280°C and the flow rate of the carrier gas (He) at 1.5 ml/min. For detection, electron impact ionization was performed. External calibration curves have been performed with a mixed of derivatized fatty acids at concentration ranging from 50 to 1000 ng/ml. Each point was injected in triplicate with SD on the measured areas below 5% for each calibration point and compounds. All calibration curves showed good linearity in the range of interest.

### TGA and DSC

TGA measurements were performed with a SDT Q600 (TA Instruments), over a temperature range of 25° to 1000°C, with a heating ramp of 5°C/min and an air flow of 75 ml/min. Sample (3 to 5 mg) was introduced into an alumina crucible with lid. Each acquisition was performed twice to ensure reproducibility of results. Data were processed using TA Universal Analysis 2000 4.5 software. DSC measurements were carried out with the same apparatus, over a temperature range from 35° to 130°C, with a ramp of 2.5°C/min under inert gas flow (N_2_) at 75 ml/min. Once heating was complete, the sample was left to cool, and a second heating ramp, identical to the first, was carried out. The second heating curve was used to determine melting transitions, as the first heating curve proved to be poorly reproducible.

## Supplementary Materials

### This PDF file includes:

Figs. S1 to S10 Table S1 References
