# Supplementary material for: Phase separation in lead-saponified drying oils: Implications for historical painting techniques and paint stability
Source: Sci Adv. 2025 Aug 27;11(35):eadt0897. doi: 10.1126/sciadv.adt0897 (PMC12383267; doi:10.1126/sciadv.adt0897)
Supplement: Supplementary file 1 — Figs. S1 to S10 Table S1 References [file sciadv.adt0897_sm.pdf]

Science Advances
AAAS

Supplementary Materials for

Phase separation in lead-saponified drying oils: Implications for historical painting techniques and paint stability

Lucie Laporte *et al.*

Corresponding author: Laurence de Viguerie, laurence.de_viguerie@sorbonne-universite.fr

Sci. Adv. **11**, eadt0897 (2025)
DOI: 10.1126/sciadv.adt0897

This PDF file includes:

Figs. S1 to S10
Table S1
References

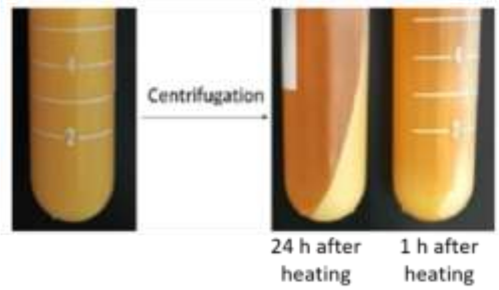

Fig. S1. Influence of time in the preparation of saponified oil. The oil was prepared by heating linseed oil with PbO 50 mol%.

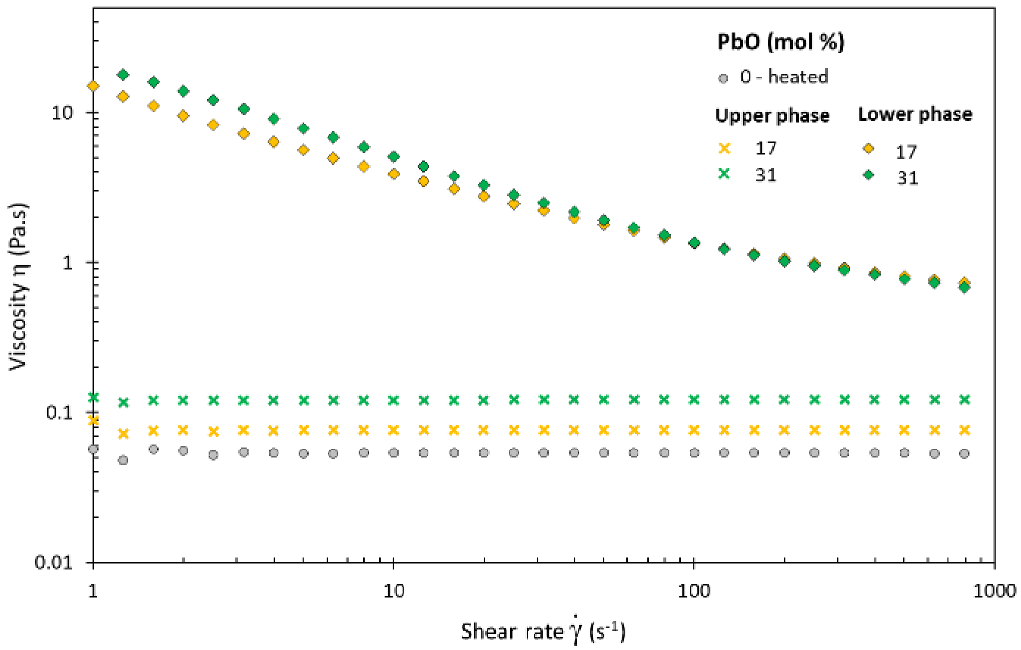

Fig. S2. Comparison of rheological properties of the two phases of other formulations. Flow tests from 1 to 1000 s^{-1} on linseed oil heated with water and linseed oil + PbO 17 and 31 mol% heated with water after centrifugation.

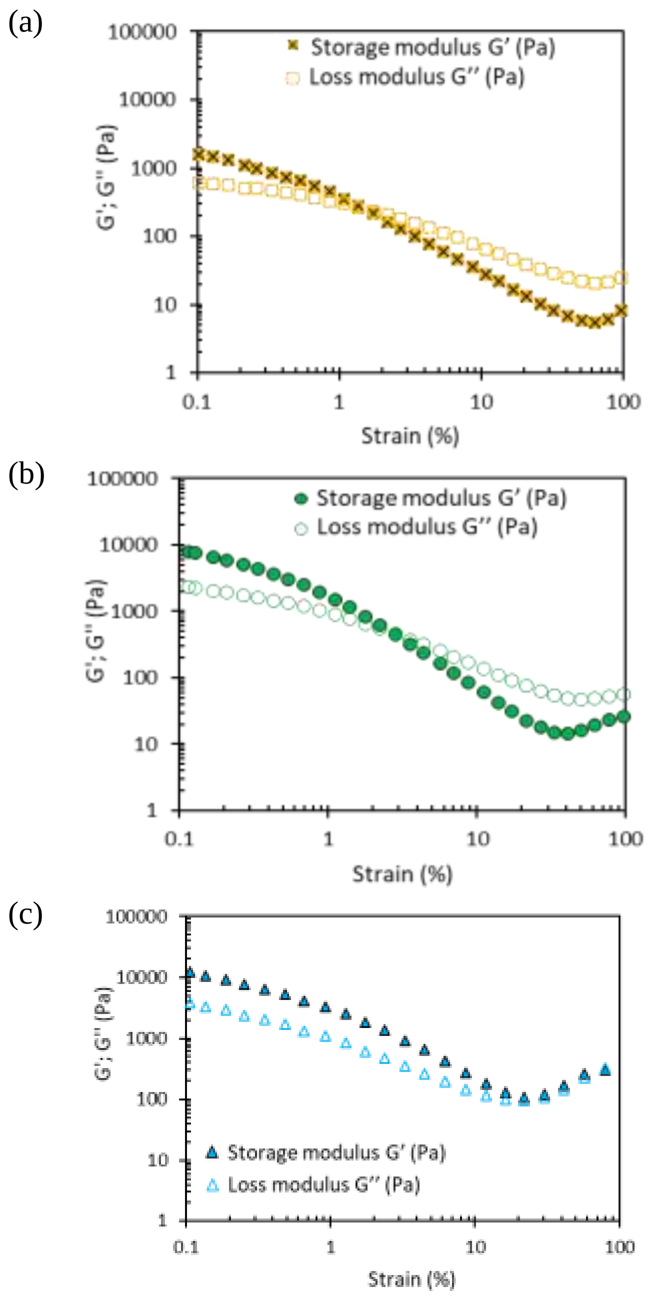

Fig. S3. Comparison of the viscoelastic properties of the lower phase of the different formulations studied. Oscillatory strain sweep test on the lower phases of linseed oil + PbO (a) 17, (b) 31 and (c) 50 mol% heated with water, from 0.05 to 1000% (f = 1 Hz).

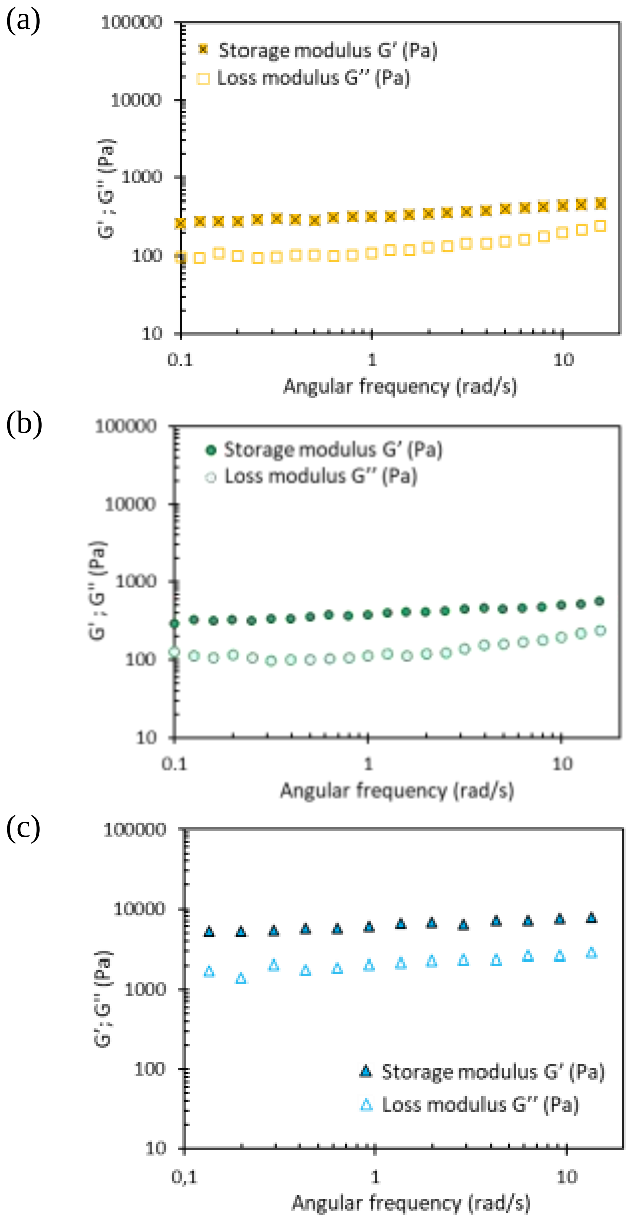

Fig. S4. Comparison of the frequency sweep tests of the lower phase of the different formulations studied. Oscillatory frequency sweep on the lower phases of linseed oil + PbO (a) 17, (b) 31 and (c) 50 mol% heated with water, from 20 to 0.1 rad s^{-1} (strain = 0.1%). Whatever the initial PbO concentration, the upper phases are viscous. The results are not shown.

(a)

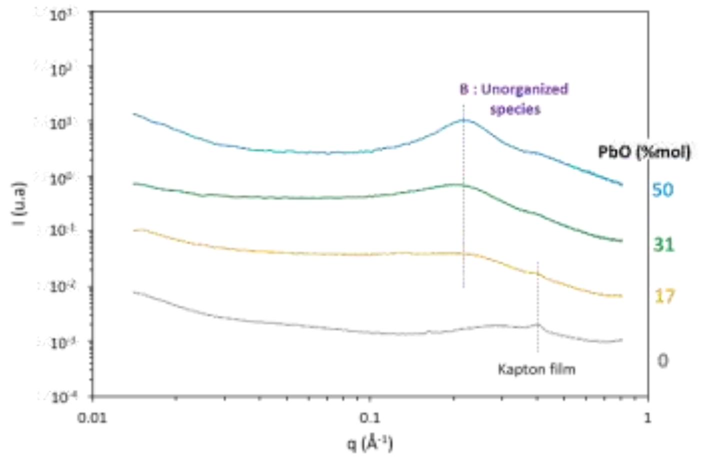

(b)

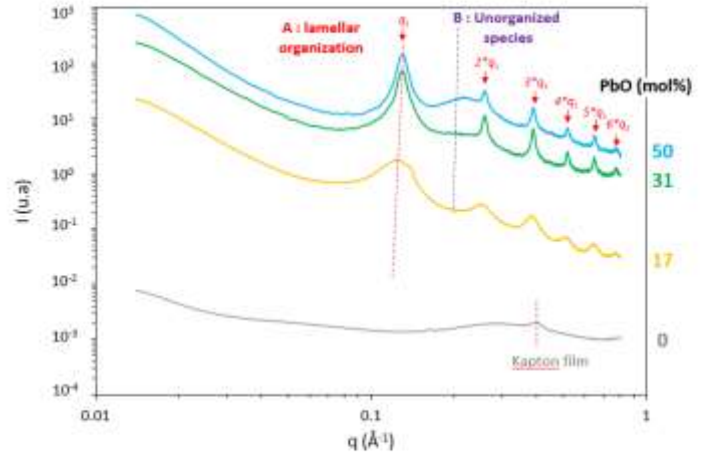

(c)

	Lower phases			Before phase separation
**mol% PbO**	17	31	50	50
**ξ (Å)**	201	663	752	567

Fig. S5. Comparison of the SAXS profiles of the two phases. Scattered intensity profiles as a function of the scattering vector *q* of upper (a) and lower (b) phases of linseed oil + PbO 17, 31 and 50 mol %, heated with water. The profile of linseed oil heated with water and without PbO is shown as a reference. The two contributions observed are noted A (lamellar organization, in red), and B (liquid order, in purple). Red arrows indicate periodic peak characteristic of local lamellar organization. (c) Correlation lengths of the lamellar domains ξ as a function of the PbO concentration, before and after phase separation. For the lower phases, the more saponified the oil, the more extended the lamellar domains. This longer-range organization suggests that species mobility is reduced in linseed oil + PbO 50 mol%, which is consistent with rheological measurements (higher viscosity and viscoelasticity).

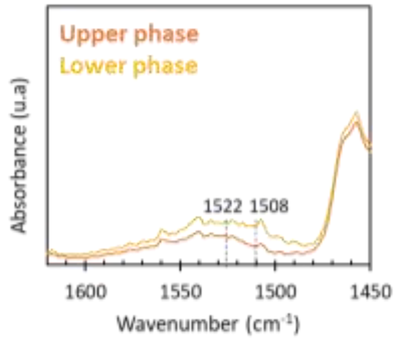

(a)

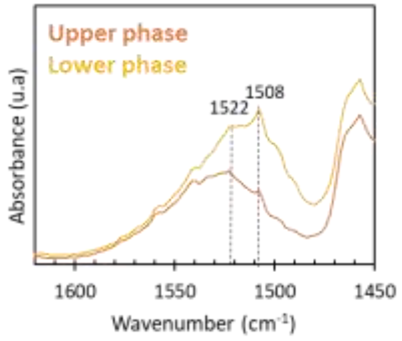

(b)

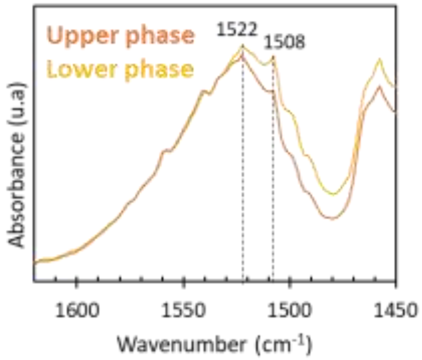

(c)

Fig. S6. Comparison of the ATR-FTIR spectra of the two phases of the different formulations studied. ATR-FTIR spectra, from 1450 to 1620 cm^{-1}, of the lower and upper phases of linseed oil samples heated with water and initially containing (a) 17, (b) 31 and (c) 50 mol% PbO. The absorption band centered at 1520 cm^{-1} corresponds to the asymmetric stretching of lead carboxylates (ν_{AS} (C=O)). The signal at 1508 cm^{-1}, more pronounced in the lower phase, has already been attributed to crystalline lead soaps *(35, 36)* and suggests that the lower phases are more ordered.

(a)

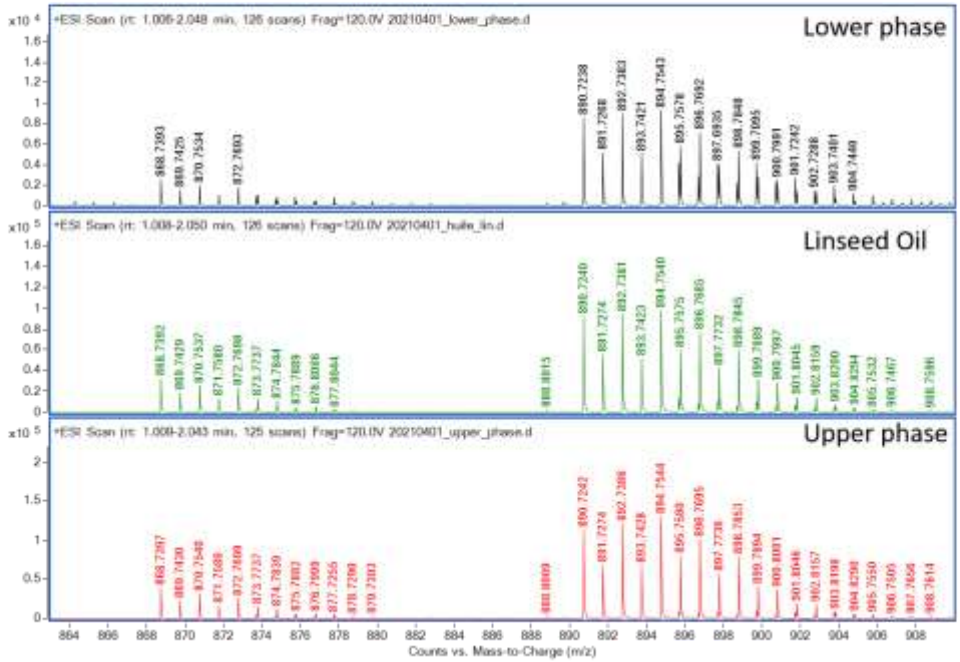

(b)

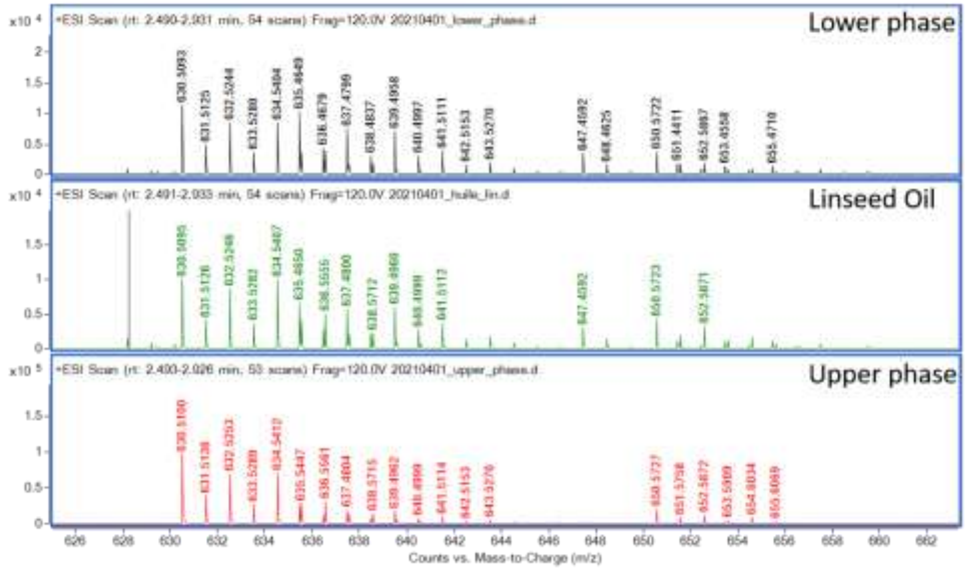

Fig. S7. Comparison of the SFC – HRMS analyses of the two phases. Positive-mode mass spectra of (a) triglycerides and (b) diglycerides from reference linseed oil and the lower and upper phases of linseed oil + PbO 50 mol% heated with water. The label *TAG 54:9 NH4*$^+$ indicates a triglyceride cationized by NH_4^+ whose aliphatic chains are composed of 54 carbon atoms and 9 unsaturations.

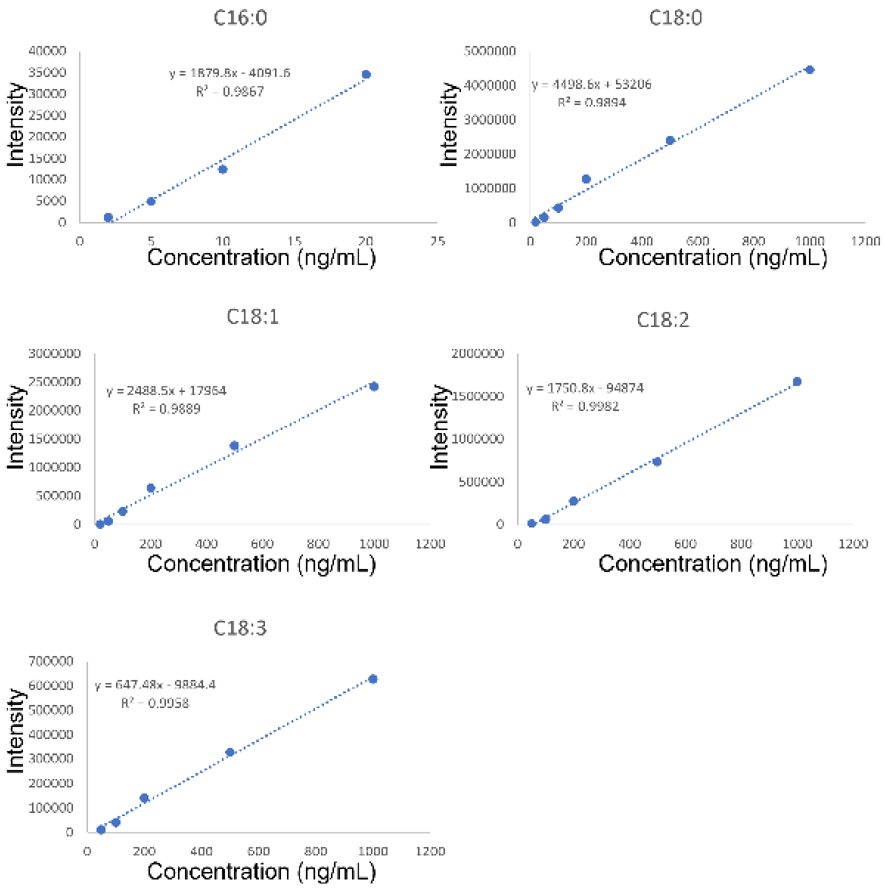

Fig. S8. Experimental calibration of GC-MS analyses. GC-MS calibration curves of palmitic (C16:0), stearic (C18:0), oleic (C18:1), linoleic (C18:2) and linolenic (C18:3) fatty acids.

Fig. S9: TGA

(a)

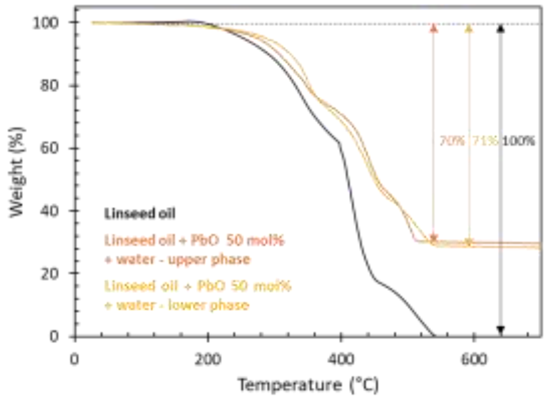

(b)

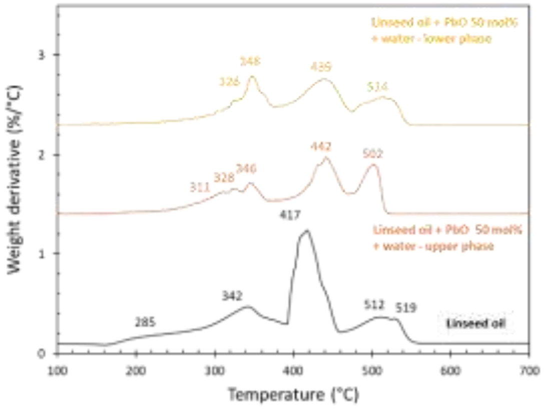

Fig. S9. Comparison of the thermogravimetric curves and their derivatives of the two phases (air flow at 5 °C/min). (a) Relative weight loss of the reference linseed oil and two phases of linseed oil + PbO 50 mol% oil + water. The given percentages correspond to the total relative mass loss over the temperature range tested, from 25 to 1000°C. (b) Weight loss derivative. Data are shifted along the y-axis for clarity. Given temperatures correspond to temperature maxima for each degradation reaction. As reported in the literature *(37)*, three different steps above 200 °C, one around 350 °C (1st stage of oxidative degradation) and two above 400 °C (main processes of decomposition) occurs in raw linseed oil. Here no weight loss was observed between 25 and 150 °C, confirming that the two phases do not contain a substantial amount of water. The same degradation steps are identified in both phases, and each degradation step induces an identical mass loss. The upper phase starts to degrade at a lower temperature than the lower phase, suggesting that it contains more species sensitive to thermo-oxidation (lower molar mass and/or unsaturated species). The main peak, at 420 °C in raw linseed oil, is shifted at higher temperature (around 440 °C) in both phases, thus indicating that they contain higher molecular mass components. In the lower phase, the peak above 500 °C is broader and at higher temperature than in the lower phase. However, the full interpretation remains tricky as this peak has not been clearly assigned.

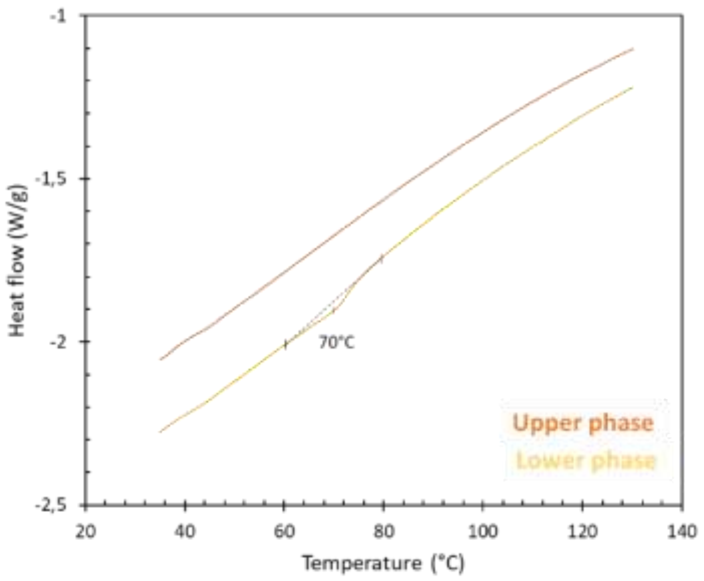

Fig. S10. Comparison of the DSC curves of the two phases (lower and upper phases of linseed oil + PbO 50 mol% heated with water). Measurements were done from 35 to 130°C, at 2.5°C/min under nitrogen flow.

**Name**	**Chemical formula**	***m/z* th ([M+NH$_4$]$^+$)**	***m/z* exp**	**Error (ppm)**
**DG 36:6**	$C_{39}H_{64}O_5$	630,5092	630,5093	0,16
**DG 36:5**	$C_{39}H_{66}O_5$	632,5249	632,5244	-0,71
**DG 36:4**	$C_{39}H_{68}O_5$	634,5405	634,5404	-0,16
**DG 36:3**	$C_{39}H_{70}O_5$	636,5562	636,5553	-1,34
**DG 36:2**	$C_{39}H_{72}O_5$	638,5718	638,5709	-1,41
**DG 36:1**	$C_{39}H_{74}O_5$			
**TG 52:3**	$C_{55}H_{94}O_6$	868,7389	868,7393	0,5
**TG 52:2**	$C_{55}H_{96}O_6$	870,7545	870,7534	-1,28
**TG 52:1**	$C_{55}H_{98}O_6$	872,7702	872,7693	-0,99
**TG 52:0**	$C_{55}H_{100}O_6$	874,7858	874,7842	-1,85
**TG 54:9**	$C_{57}H_{92}O_6$	890,7232	890,7238	0,66
**TG 54:8**	$C_{57}H_{94}O_6$	892,7389	892,7383	-0,63
**TG 54:7**	$C_{57}H_{96}O_6$	894,7545	894,7543	-0,24
**TG 54:6**	$C_{57}H_{98}O_6$	896,7702	896,7692	-1,08
**TG 54:5**	$C_{57}H_{100}O_6$	898,7858	898,7848	-1,13
**TG 54:4**	$C_{57}H_{102}O_6$	900,8015	900,7991	-2,63
**TG 54:3**	$C_{57}H_{104}O_6$	902,8171	902,8153	-2,01

Table S1. SFC peaks annotations (related to Figure S7).

REFERENCES AND NOTES

1. S. Zumbühl, *Historical Siccatives for Oil Paint and Varnishes - The Use of Lead Oxide, Alum, White Vitriol, Pumice, Bone Ash and Venetian Glass as Driers: Historical Written Sources - Production and Raw Material Quality - Technological Significance* (HDW Publications, 2022).

2. T. T. de Mayerne, M. Faidutti, C. Versini, Pictoria Sculptoria et Quae Subalternarum Artium: 1620 (Audin Imprimeurs, 1967). [Painting, Sculpture, and the Subordinate Arts].

3. L. de Viguerie, P. A. Payard, E. Portero, P. Walter, M. Cotte, The drying of linseed oil investigated by Fourier transform infrared spectroscopy: Historical recipes and influence of lead compounds. *Prog. Org. Coat.* **93**, 46–60 (2016).

4. M. R. Mills, *An Introduction to Drying Oil Technology* (Pergamon, 1952).

5. J. D. J. van den Berg, "Analytical chemical studies on traditional linseed oil paints," thesis, University of Amsterdam, Amsterdam (2002).

6. J. C. Martin, M. Nour, F. Lavillonnière, J. L. Sébédio, Effect of fatty acid positional distribution and triacylglycerol composition on lipid by-products formation during heat treatment: II. *Trans* isomers. *J. Am. Oil Chem. Soc.* **75**, 1073–1078 (1998).

7. J. C. Martin, M. C. Dobarganes, M. Nour, G. Marquez-Ruiz, W. W. Christie, F. Lavillonnière, J. L. Sébédio, Effect of fatty acid positional distribution and triacylglycerol composition on lipid by-products formation during heat treatment: I. Polymer formation. *J. Am. Oil Chem. Soc.* **75**, 1065–1071 (1998).

8. J. D. J. van den Berg, N. D. Vermist, L. A. Carlyle, M. Holcapek, J. B. Boon, Effects of traditional processing methods of linseed oil on the composition of its triacylglycerols. *J. Sep. Sci.* **27**, 181–199 (2004).

9. M. Cotte, E. Checroun, J. Susini, P. Dumas, P. Tchoreloff, M. Besnard, P. Walter, Kinetics of oil saponification by lead salts in ancient preparations of pharmaceutical lead plasters and painting lead mediums. *Talanta* **70**, 1136–1142 (2006).

10. L. de Viguerie, G. Ducouret, M. Cotte, F. Lequeux, P. Walter, New insights on the glaze technique through reconstruction of old glaze medium formulations. *Colloids Surf.* **331**, 119–125 (2008).

11. L. Laporte, G. Ducouret, F. Gobeaux, A. Lesaine, C. Hotton, T. Bizien, L. Michot, L. de Viguerie, Rheo-SAXS characterization of lead-treated oils: Understanding the influence of lead driers on artistic oil paint's flow properties. *J. Colloid Interface Sci.* **633**, 566–574 (2023).

12. L. Laporte, F. Gobeaux, T. Pouget, N. Benoot, J. Foisnon, D. Touboul, G. Ducouret, L. de Viguerie, Multiscale organisation of lead carboxylates in artistic oil binders. *Phys. Chem. Chem. Phys.* **26**, 2657–2665 (2024).

13. M. J. Plater, B. M. de Silva, T. Glebrich, M. B. Hursthouse, C. L. Higgitt, D. R. Saunders, The characterisation of lead fatty acid soaps in 'protrusions' in aged traditional oil paint. *Polyhedron* **22**, 3171–3179 (2003).

14. R. M. A. Heeren, J. J. Boon, P. Noble, J. Wadum, "Integrating imaging FTIR and secondary ion mass spectrometry for the analysis of embedded paint cross-sections," in *ICOM-CC Triennial Meeting (12th), Lyon* (James & James, 1999), pp. 228–233.

15. V. Gonzalez, M. Cotte, G. Wallez, A. van Loon, W. de Nolf, M. Eveno, K. Keune, P. Noble, J. Dik, Unraveling the composition of Rembrandt's impasto through the identification of unusual plumbonacrite by multimodal x-ray diffraction analysis. *Angew. Chem. Int. Ed. Engl.* **58**, 5619–5622 (2019).

16. V. Gonzalez, G. Wallez, E. Ravaud, M. Eveno, I. Fazlic, T. Fabris, A. Nevin, T. Calligaro, M. Menu, V. Delieuvin, M. Cotte, X-ray and infrared microanalyses of Mona Lisa's ground layer and significance regarding Leonardo da Vinci's palette. *J. Am. Chem. Soc.* **145**, 23205–23213 (2023).

17. H. P. Klug, L. E. Alexander, *X-Ray Diffraction Procedures: For Polycrystalline and Amorphous Materials* (Wiley, ed. 2, 1974).

18. N. C. Acevedo, A. G. Marangoni, Characterization of the nanoscale in triacylglycerol crystal networks. *Cryst. Growth Des.* **10**, 3327–3333 (2010).

19. C. R. Safinya, D. Roux, G. S. Smith, S. K. Sinha, P. Dimon, N. A. Clark, A. M. Bellocq, Steric interactions in a model multimembrane system: A synchrotron x-ray study. *Phys. Rev. Lett.* **57**, 2718–2721 (1986).

20. F. Castro-Roman, L. Porcar, G. Porte, C. Ligoure, Quantitative analysis of lyotropic lamellar phases SANS patterns in powder-oriented samples. *Eur. Phys. J. E Soft Matter* **18**, 259–272 (2005).

21. I. W. Hamley, Diffuse scattering from lamellar structures. *Soft Matter* **18**, 711–721 (2022).

22. M.-C. Corbeil, L. Robinet, X-ray powder diffraction data for selected metal soaps. *Powder Diffr.* **17**, 52–60 (2002).

23. F. Martínez-Casado, J. A. Rodríguez-Cheda, M. Ramos-Riesco, M. Redondo-Yélamos, F. Cucinotta, A. Fernández-Martínez, "Physicochemistry of pure lead (ii) soaps: Crystal structures, solid and liquid mesophases, and glass phases – Crystallographic, calorimetric, and pair distribution function analysis," in *Metal Soaps in Art: Conservation and Research* (Springer, 2019), pp. 227–239.

24. C. Himawan, V. M. Starov, A. G. F. Stapley, Thermodynamic and kinetic aspects of fat crystallization. *Adv. Colloid Interface Sci.* **122**, 3–33 (2006).

25. M. Kellens, W. Meeussen, H. Reynaers, Study of the polymorphism and the crystallization kinetics of tripalmitin: A microscopic approach. *J. Am. Oil Chem. Soc.* **69**, 906–911 (1992).

26. A. G. Marangoni, M. Ollivon, Fractal character of triglyceride spherulites is a consequence of nucleation kinetics. *Chem. Phys. Lett.* **442**, 360–364 (2007).

27. M. G. MacDonald, M. R. Palmer, M. R. Suchomel, B. H. Berrie, Reaction of Pb(II) and Zn(II) with ethyl linoleate to form structured hybrid inorganic-organic complexes: A model for degradation in historic paint films. *ACS Omega* **1**, 344–350 (2016).

28. C. Chollet, S. Boutet-Mercey, L. Laboureur, C. Rincon, M. Méjean, J. Jouhet, F. Fenaille, B. Colsch, D. Touboul, Supercritical fluid chromatography coupled to mass spectrometry for lipidomics. *J. Mass Spectrom.* **54**, 791–801 (2019).

29. O. Kozlov, E. Hančová, E. Cífková, M. Lísa, Comprehensive single-platform lipidomic/metabolomic analysis using supercritical fluid chromatography-mass spectrometry. *Anal. Chem.* **96**, 1320–1327 (2024).

30. J. Zhang, Y. Gao, M. Zhao, X. Xu, B. Xi, L. Lin, J. Zheng, B. Chen, Y. Shu, C. Li, Y. Shen, Detection of walnut oil adulterated with high-linoleic acid vegetable oils using triacylglycerol pseudotargeted method based on SFC-QTOF-MS. *Food Chem.* **416**, 135837 (2023).

31. J. La Nasa, A. Lluveras-Tenorio, F. Modugno, I. Bonaduce, Two-step analytical procedure for the characterization and quantification of metal soaps and resinates in paint samples. *Herit. Sci.* **6**, 57–67 (2018).

32. G. Knothe, R. Dunn, A comprehensive evaluation of the melting points of fatty acids and esters determined by differential scanning calorimetry. *J. Am. Oil Chem. Soc.* **86**, 843–856 (2009).

33. P. Noble, "A brief history of metal soaps in paintings from a conservation perspective," in *Metal Soaps in Art: Conservation and Research* (Springer, 2019), pp. 1–22.

34. J. Maroger, *A la recherche des secrets des grands peintres* (Dessain et Tolra, 1986).

35. J. Catalano, A. Murphy, Y. Yao, G. P. A. Yap, N. Zumbulyadis, S. A. Centeno, C. Dybowski, Coordination geometry of lead carboxylates – Spectroscopic and crystallographic evidence. *Dalton Trans.* **44**, 2340–2347 (2015).

36. J. Hermans, L. Zuidgeest, P. Iedema, S. Woutersen, K. Keune, The kinetics of metal soap crystallization in oil polymers. *Phys. Chem. Chem. Phys.* **23**, 22589–22600 (2021).

37. I. Bonaduce, L. A. Carlyle, M. P. Colombini, C. Duce, C. Ferrari, E. Ribechini, P. Selleri, M. R. Tiné, New insights into the ageing of linseed oil paint binder: A qualitative and quantitative analytical study. *PLOS ONE* **7**, e49333 (2012).
